# Oxidative Stress, Neuroinflammation, and NADPH Oxidase: Implications in the Pathogenesis and Treatment of Alzheimer's Disease

**DOI:** 10.1155/2021/7086512

**Published:** 2021-04-16

**Authors:** Upasana Ganguly, Upinder Kaur, Sankha Shubhra Chakrabarti, Priyanka Sharma, Bimal Kumar Agrawal, Luciano Saso, Sasanka Chakrabarti

**Affiliations:** ^1^Department of Biochemistry, MM Institute of Medical Sciences & Research, Maharishi Markandeshwar Deemed University, Mullana, Ambala, Haryana, India; ^2^Department of Pharmacology, Institute of Medical Sciences, Banaras Hindu University, Varanasi, India; ^3^Department of Geriatric Medicine, Institute of Medical Sciences, Banaras Hindu University, Varanasi, India; ^4^Department of General Medicine, MM Institute of Medical Sciences & Research, Maharishi Markandeshwar Deemed University, Mullana, Ambala, Haryana, India; ^5^Department of Physiology and Pharmacology “Vittorio Erspamer”, Sapienza University of Rome, Rome, Italy

## Abstract

NADPH oxidase as an important source of intracellular reactive oxygen species (ROS) has gained enormous importance over the years, and the detailed structures of all the isoenzymes of the NADPH oxidase family and their regulation have been well explored. The enzyme has been implicated in a variety of diseases including neurodegenerative diseases. The present brief review examines the body of evidence that links NADPH oxidase with the genesis and progression of Alzheimer's disease (AD). In short, evidence suggests that microglial activation and inflammatory response in the AD brain is associated with increased production of ROS by microglial NADPH oxidase. Along with other inflammatory mediators, ROS take part in neuronal degeneration and enhance the microglial activation process. The review also evaluates the current state of NADPH oxidase inhibitors as potential disease-modifying agents for AD.

## 1. Introduction

A review on oxidative stress and disease mechanisms and therapeutic use of antioxidants is usually not greeted with much enthusiasm, and reasons are not difficult to surmise. The story of oxidative stress and its involvement in multiple disease mechanisms and aging is quite old, spanning many decades [1]. However, a definitive evidence of oxidative stress as a driving mechanism of disease pathogenesis is still lacking. Likewise, inconsistent results of multiple clinical trials of antioxidants in various diseases, especially in neurodegenerative diseases, have been a cause for great disappointment to the advocates of free radical hypothesis of diseases [2–4]. On the other hand, there is a considerable body of experimental evidence that suggests the involvement of reactive oxygen species (ROS) and reactive nitrogen species (RNS), generally free radicals of oxygen and nitrogen, in the pathophysiology of many diseases and aging [4–6]. Thus, the free radical biology in health and disease has grown gradually over the years from the identification of various species of ROS and RNS in living systems and the elucidation of the chemistry and kinetics of their interactions to the identification of their toxic effects on different biomolecules and cell organelles [7, 8]. Further, the enzymatic or nonenzymatic antioxidant defense system of the body and the redox-signalling pathways regulating physiological and pathological processes within the cells have been identified [7, 9, 10]. The redox-signalling pathways play important roles in cell growth, differentiation, and death as evidenced from a plethora of experimental studies [10–12].

The antioxidant defense of the body generally counterbalances the various ROS and RNS in the normal physiological condition, but in pathological conditions when the free radicals overpower the antioxidant defense, a state of oxidative stress develops. It is presumable that direct damage to biomolecules within the cells by ROS and RNS and aberrant redox-signalling pathways together are involved in many diseases including neurodegenerative disorders [5, 6, 13, 14]. The new technique of redox proteomics with the availability of antibodies against redox-modified proteins has strengthened our understanding of oxidative stress-induced mechanisms in diseases [15]. On the other hand, the identification of new antioxidants from natural sources or synthesis of novel, multifunctional, and organelle-targeted antioxidants has provided a new impetus in therapeutic applications of antioxidants [16–19]. So far, antioxidants have been developed with the precise aim of scavenging the free radicals of oxygen to prevent the deleterious effects of oxidative stress. Much less attempt has been made to prevent the generation of ROS at the source presumably because ROS are generated in most cases as a by-product of important metabolic pathways like the electron transport chain (ETC) of mitochondria which precludes the option of inhibiting the generation of ROS at the source. However, NADPH oxidase (NOX) is one enzyme whose sole function is the production of reactive oxygen species like superoxide radical (O_2_^•-^) and hydrogen peroxide, and therefore, inhibition of NOX could be an important rescue avenue against oxidative stress in tissues in different disease conditions. Thus, the therapeutic potential of NOX inhibitors should be explored thoroughly by experimental and clinical research.

## 2. ROS and NADPH Oxidase

### 2.1. Sources of ROS

The sources of ROS within the cells are varied, but they predominantly occur as a by-product of enzymatic reactions in different metabolic pathways. The ETC of mitochondria is a major contributor of intracellular ROS, but many other enzyme complexes in mitochondria like pyruvate dehydrogenase, *α*-ketoglutarate dehydrogenase, *cis*-aconitase, glycerophosphate dehydrogenase, and dihydroorotate dehydrogenase are also responsible for ROS production [20–22]. Of the various complexes of ETC, complex I and complex III are the major sites of ROS production. The enzymes like cyclooxygenase, lipoxygenase, xanthine oxidase, and cytochrome P450-dependent oxygenases also contribute to ROS production [22]. Nonenzymatic or enzymatic oxidation of catecholamines and autoxidation of hemoglobin can also produce ROS under physiological conditions. However, another major source of ROS production is NOX, which was initially identified in neutrophils as the enzyme responsible for the production of superoxide radicals during a “respiratory burst” [23]. Since then, the biochemistry of NOX has been extensively studied, and this enzyme (in various isoforms) is present in a variety of tissues including the brain as well as in multiple cell lines. The NOX family of enzymes is comprised of 7 isoenzymes named as NOX1-5 and dual oxidases DUOX1 and DUOX2. NOX is responsible for the production of superoxide radicals (O_2_^**•-**^) by the transfer of electrons to molecular oxygen from NADPH via FAD and two heme residues of the enzyme. The O_2_^•-^ radicals in turn undergo dismutation to produce H_2_O_2_, but it has been suggested that the isoenzymes NOX4, DUOX1, and DUOX2, could directly produce H_2_O_2_ [23, 24].

### 2.2. NADPH Oxidase: Structure and Isozymes

Each of the seven members of the NOX family has a catalytic subunit comprised of a transmembrane domain of 6 *α*-helical segments containing two heme units liganded to histidine residues and a cytosolic segment which contains FAD and NADPH binding sites [23, 24]. Additional structural features of the catalytic subunit are seen in some of the isoenzymes such as the EF-calcium-binding domains of NOX5, DUOX1, and DUOX2 and peroxidase domains of DUOX enzymes [24, 25]. This large membrane-bound (plasma membrane and membranes of some organelles) subunit of NOX is associated with another membrane-bound protein called p22^phox^ in some of the isoenzymes like NOX1, NOX2, NOX3, and NOX4, and the large heterodimer thus formed is called flavocytochrome b558. Likewise, the catalytic subunit of DUOX1 or DUOX2 is associated with another membrane-embedded protein called DUOXA1 or DUOXA2, respectively [24]. In addition, the NOX family of enzymes requires several other regulatory cytosolic proteins for activation, stability, or full function, and these additional interaction proteins are different for different isoenzymes [23, 24]. NOX2, which was the first isoenzyme to be identified in phagocytic cells, has been studied most, and its activation process may be taken as the prototype for other NOX family of enzymes. The catalytic subunit of NOX2 is known as gp91^phox^ which remains in association with a membrane-embedded protein called p22^phox^ which confers stability to the complex. During agonist activation, the cytosolic protein p47^phox^ is phosphorylated, which in association with some other cytosolic proteins called p67^phox^ and p40^phox^ and GTP-binding proteins like Rac2 and Rap1A translocates to the membrane to interact with the heterodimer of gp91^phox^ and p22^phox^ forming a fully functional enzyme. The interaction of src-homology domain 3 (SH3) of p47^phox^ with the C-terminal segment of p22^phox^, at a proline-rich sequence, is important in this assembly [23–25]. The detailed molecular interactions of these proteins during NOX2 activation and the functions of individual proteins have been identified. For example, p47^phox^ is considered as an organizer protein without any catalytic property, while the activator protein p67^phox^ increases the catalytic activity of NOX2. For some other NOX isoenzymes like NOX1, NOX3, and NOX4, a similar pattern of activation with some variations is seen with the aid of multiple regulatory proteins (activators or organizers) like NOXO1, NOXA1, PDI, and Poldip2, as well several GTP-binding proteins [25, 26]. NOX5, DUOX1, and DUOX2, however, do not need any such assistance from other proteins, and they are presumably activated by the binding of Ca^2+^ ions at their cytosolic EF-calcium-binding domains [25, 26].

### 2.3. NADPH Oxidase: Physiological Role and Pathological Implications

In contrast to other sources, NOX is distinctive in producing ROS through a highly regulated complex enzymatic process and not as a by-product of a main reaction. Thus, the physiological role of NOX-dependent ROS production needs to be carefully analysed. In general, NOX-dependent ROS takes part in redox-signalling pathways through redox-responsive signalling molecules (transcription factors, soluble or receptor-kinases, etc.), and the process regulates various aspects of cell growth, differentiation, survival, and metabolism with broad implications in immunity and inflammation, aging, cancer, and cardiovascular function and malfunctions [23, 24, 27–29]. For example, multiple studies have shown that vascular endothelial function and migration, angiogenesis, expression of cell adhesion molecules, vascular smooth muscle cell proliferation, etc. are regulated by multiple agonists like angiotensin II, growth factors, and cytokines through modulation of NADPH-dependent ROS production [28–30]. NOX-dependent ROS signalling is also involved in regulating cardiac remodelling, cardiomyocyte hypertrophy, and interstitial fibrosis after myocardial infarction or during chronic cardiac stress from hypertension [31]. The NOX4 isozyme, expressed in the mitochondria of cardiomyocytes, is particularly important in this context as has been shown in an experimental study; in heart-specific NOX4 knock-out mice, cardiac hypertrophy, interstitial fibrosis, mitochondrial dysfunction, and apoptosis of cardiomyocytes following pressure overload are significantly prevented compared to that in wild-type mice [32]. In cardiac-specific human NOX4 transgenic mice (hNOX4), a significant overexpression of NOX4 was observed with a high level of ROS production and associated myocardial fibrosis under basal condition when compared to littermate controls negative for hNOX4 [33]. This study further demonstrated that treatment with angiotensin II caused cardiac hypertrophy and myocardial fibrosis along with NOX4 upregulation and ROS production in control mice, and all these changes were much more aggravated in hNOX4 [33]. NOX4 in cardiomyocytes has been shown to be regulated by a tyrosine kinase belonging to Src-family [34].

NOX-generated ROS play an important role in tumor cell proliferation, metabolism, and progress. A systematic review has shown the association of lung cancer with increased expression and activity of NOX in tumor tissue and that inhibition of NOX could prevent tumor progression during *in vitro* experiments [35]. Similarly, overexpression of NOX2 has been reported in a significant number of human gastric carcinoma cases along with increased expression of EGFR and VEGF suggesting a biomarker potential of the former in this cancer [36]. Another study demonstrated a dysregulation of the expression of the NOX family of isoenzymes in human gastric cancer with overexpression of NOX2 indicating better prognosis and elevated NOX4 and decreased DUOX1 associated with worse outcome [37]. In other studies with human cancer tissue or cancer cell lines, the NOX family of isoenzymes is expressed differentially that probably has implications in tumor growth and invasion [38]. It is still debatable how exactly NOX regulates tumor growth and progression, but genomic instability caused by ROS, inactivation of p53 function, alterations in the functions of cell signalling kinases and phosphatases, and modulation of the functions of the *ras* oncogene may all be contributing to this process [39, 40]. Another important function of NOX-mediated ROS is to modulate innate immunity and the inflammatory response. The role of NOX is well established in the respiratory burst of neutrophils, which is a component of innate immunity and a first-line defense against invading microbes. The other components of innate immunity involve pattern recognition receptors like membrane-bound toll-like receptors (TLRs) and several cytosolic receptors. These pattern recognition receptors respond to specific patterns in the bacterial proteins, DNA, peptidoglycan, lipopolysaccharide (LPS) etc. or DNA or RNA viruses and initiate multiple signalling mechanisms for host defense [41, 42]. The involvement of NOX-derived ROS has been shown to be necessary for TLR-2-dependent innate immune response against *Mycobacterium tuberculosis* [43]. Similarly, NOX-dependent ROS generation is necessary for RIG-I-mediated activation of the transcription factor IRF-3 leading to antiviral gene expression [44]. The transcription factor NF-*κ*B is involved in the expression of multiple genes related to innate immunity, and bacterial LPS-induced activation of NF-*κ*B through TLR-4 requires NOX-dependent ROS production [45]. Although NOX-mediated ROS production is important for host defense against microbial invasion, the role of NOX is possibly more complex. For example, influenza-A-induced lung damage is aggravated by NOX2 isoenzymes, while DUOX2 isoenzymes are apparently protective [46]. Apart from influenza virus, other respiratory viruses like human respiratory syncytial virus and human rhinovirus may also cause increased ROS production by different isoenzymes of NOX, and despite the important role of NOX in host defense, NOX-derived ROS could aggravate lung inflammation and damage in many cases. Under such circumstances, the use of NOX inhibitors may become a potential therapeutic option [46, 47].

The role of oxidative damage in aging has been suggested many decades ago, and the original concept of progressive accumulation of oxidatively damaged biomolecules within cells with functional deterioration of tissues was quite straightforward [48, 49]. Thus, oxidative damage markers have been shown to accumulate in different tissues of aged animals in multiple studies, and ROS derived from mitochondrial metabolism and NOX have been both implicated in this process [48, 50–52]. The precise role of NOX in age-related oxidative damage in brain has been shown using NOX2 knock-out mice or transgenic mice overexpressing NOX2 [53]. Age-dependent increase in NOX activity has been shown in the aged brain of rodents which is modulated by dietary manipulations [54, 55]. Likewise, NOX4 upregulation has been implicated in aging of heart [56]. However, most of these studies indicating a relationship of oxidative stress and aging in mammals are actually correlative in nature and do not necessarily identify a causal relationship. Moreover, this simple concept of generalized and indiscriminate ROS-mediated damage to tissues as a pivotal mechanism of aging seemed contradictory when genetic manipulation studies overexpressing or knocking out antioxidant enzyme genes or environmental modifications in nematodes, flies, yeast, and other organisms revealed that complex interactions of ROS with life span extension genes instead of direct oxidative damage to tissue components are involved in aging and alteration of the life span of these species [57–61]. Both mitochondrial- and NOX-derived ROS at moderate levels are involved in actually increasing the longevity of these organisms by redox-signalling pathways, while presumably at higher levels of ROS, both oxidative damage and shortening of life span occur [59, 62–64]. However, the results of such life span alteration experiments on mammals by knocking out antioxidant enzyme genes or NOX have remained controversial, failing to show clearly the link between oxidative stress and longevity [65–68]. The redox-signalling pathways comprised of various redox-sensitive transcription factors and cell signalling kinases and their downstream components have also been identified and explored in detail in mammalian cells, but their precise role in mammalian aging at the organismal level has not been elucidated yet [10, 49, 69].

### 2.4. NADPH Oxidase in the Brain

The vulnerability of the brain to oxidative damage is a well-accepted fact based on high oxygen consumption of the organ, availability of transition metals and autooxidizable catecholamines, abundance of polyunsaturated fatty acids, and the presence of a relatively weak antioxidant defense [13, 70]. Oxidative stress is implicated in many diseases of the central nervous system (CNS); thus, NOX has been studied in a variety of pathological conditions of the brain [71, 72]. The four NOX isoenzymes (NOX1, NOX2, NOX3, and NOX4) in the brain have been well explored in the context of brain development, aging and pathological conditions like ischemic or traumatic injury of the brain, neurodegenerative diseases, and different types of psychosis, but less information is available on NOX5 or the DUOX enzymes [72–76]. Although several early studies identified NOX2 in microglia, the inflammatory cells of the brain, many studies subsequently demonstrated the involvement of neuronal, astrocytic, or brain endothelial NOX in different conditions [72, 74, 75, 77]. NOX4 mRNA was shown to be overexpressed in neurons and newly formed capillaries in the brain in an experimental mouse model of ischemia, which persisted up to one month after the onset of ischemia [78]. Using a luminometric enzyme assay, NOX was also shown to be elevated in aged rat brain associated with increased accumulation of proinflammatory cytokines [55]. The age-dependent increase in ROS production and loss of neurons and capillaries in the brain of aged rats compared to young animals were shown to be significantly decreased in NOX2 knock-out rats [53]. In patients dying of traumatic brain injury (TBI), a selective increase in NOX2 was observed in parvalbumin-positive interneurons, but not in microglia, of postmortem brain without much changes in NOX1 and NOX4 isoenzymes as studied by immunohistochemistry [79].

NOX is also expressed in various primary cultures of neurons or neural stem cells or neural cell lines, and these have been manipulated genetically or pharmacologically to gain insight into normal or toxic functions of NOX-derived ROS in neurons. Thus, in primary culture of cerebellar granule cells, NOX is overexpressed in growth cones and filopodia with NOX-derived ROS involved in neuronal maturation [80]. Similarly, in primary culture of rat hippocampal neurons, genetic or pharmacological inactivation of NOX has been shown to cause altered neuronal polarization and inhibition of axonal growth [81]. In neural stem cells derived from mouse embryonic hippocampus, NOX inhibitors or ROS scavengers can inhibit cellular proliferation [82]. In neural crest stem cells, neuronal differentiation induced by bone morphogenetic protein 2 (BMP-2) in culture is regulated by NOX4 isoenzymes [83]. Likewise, nerve growth factor-induced differentiation of PC12 cells to a neuronal phenotype requires the presence of NADPH-dependent ROS [84]. Furthermore, NOX-dependent ROS have been suggested to play a crucial signalling role in long-term potentiation (LTP) and synaptic plasticity in several studies using pharmacological inhibition of NOX or by employing transgenic mice without functional NOX activity [85]. On the other hand, apoptosis of sympathetic neurons in culture under condition of nerve growth factor deprivation could be prevented by the inhibitor of NOX, suggesting a role of ROS in programmed cell death [86]. Overall, these studies indicate that ROS derived from NADPH oxidase are implicated in a multitude of signalling functions within neurons in normal conditions while their deleterious effects could be important in diseased conditions.

### 2.5. Oxidative Stress, NADPH Oxidase, and Neurodegenerative Diseases

Oxidative stress is an important element in the pathogenesis of many neurodegenerative diseases, where the deleterious actions of ROS on critical cellular components like proteins, phospholipids, and DNA could lead to the disruption and dysfunction of cell physiology which could be important in the genesis or progression of the disease [87–89]. The direct damage to cellular components by ROS has been extensively studied *in vitro*, and the pathways and end-products of proteins, phospholipids, and DNA oxidation have been elaborately identified forming a vast mass of literature on free radical biology [90–92]. The end-products of such oxidative damage pathways often accumulate in high amounts in many pathological conditions in the tissues and body fluids, which are measured as oxidative damage markers, and this is generally thought to be indicative of oxidative stress in pathological conditions. Thus, accumulation of phospholipid damage markers like malondialdehyde (MDA) or 4-hydroxynonenal (HNE) or F2 isoprostanes; protein damage markers like protein carbonyls and HNE protein adducts; or DNA damage markers like 8-hydroxydeoxyguanosine (8-OHdG) have been demonstrated in the brain, cerebrospinal fluid (CSF), and blood in many neurodegenerative diseases [93–97]. In contrast to such indiscriminate damage to cellular components by ROS, a regulated cell death process called ferroptosis has recently been identified, which is iron-dependent and requires ROS and lipid peroxidation products [98]. The morphological and biochemical characteristics of ferroptosis have been worked out, which appear to be different from apoptosis and necroptosis, and the process is triggered by a diverse group of molecules like erastin, sulphasalazine, BSO, DP12, DP17, cisplatin, and glutamate, many of which deplete the intracellular level of reduced glutathione (GSH) or inhibit glutathione peroxidase 4 (GPX4) [98, 99]. The biochemical features include increased production of ROS, accumulation of lipid peroxidation products, elevated intracellular level of iron, decreased glutathione level, and inhibition by iron-chelators and lipid-soluble antioxidants like *α*-tocopherol, ferrostatin-1, and liproxstatin-1. The most characteristic morphological feature is the presence of deformed, shrunken mitochondria with loss of cristae and ruptured outer membrane and an intact nucleus [98–100]. The process of ferroptosis has been implicated in several pathological conditions like acute kidney injury, ischemia-reperfusion injury, cancer, and several neurodegenerative diseases [98, 100]. In addition to these mechanisms, ROS have been shown to be involved both in apoptosis and regulated necrosis (necroptosis) in many experimental conditions, and this could be important in the context of neuronal death which is a hallmark feature of most neurodegenerative diseases [101–103].

Though the involvement of ROS in various cell death pathways is complex, contextual, and probably interrelated, it may be presumed that as a major contributor to intracellular ROS production, NOX would play an important role in cellular death pathways. Thus, enhanced NADPH oxidase activity has been reported in apoptosis and necroptosis in a variety of experimental models involving cardiomyocytes, pancreatic acinar cells, human aortic smooth muscle cells, endothelial cells, and fibroblasts [104–108]. In the context of neuronal death in AD, we will discuss the role of NOX separately, but there is scattered evidence of NOX activation in other neurodegenerative diseases as well. Thus, postmortem brain studies have revealed high levels of NOX2 in the substantia nigra of sporadic Parkinson's disease (PD) patients, localizing with the microglial marker CD68 as evidenced by immunostaining [109]. In the same study, high levels of NOX2 in reactive microglia associated with dopaminergic neuronal loss have been observed in 1-methyl-4-phenyl-1,2,3,6-tetrahydropyridine- (MPTP-) induced experimental models of PD, and interestingly, much less dopaminergic neuronal loss is noticed in mice lacking NOX2 suggesting a clear link between neuronal death and NOX2 activation [109]. In another study of a 6-hydroxydopamine-based model of PD neurodegeneration in rats, increased expression levels of NOX1 and Rac1 (a component of the NOX1 complex) have been observed in dopaminergic neurons of the substantia nigra along with oxidative DNA damage and neuronal death which could be prevented significantly by knocking down the expression of NOX1 [110]. Oxidative stress and NOX have been implicated in the pathogenesis of another devastating neurodegenerative disease—amyotrophic lateral sclerosis (ALS)—in which progressive loss of motor neurons accompanied by gliosis occurs. In spinal cords of genetic mouse models of ALS, an increased expression of NOX2 has been demonstrated, and a similar increase has been observed in microglial NOX2 in postmortem spinal cord samples of ALS patients [111]. This study, however, has failed to show any improvement in the survival of ALS mouse models upon treatment with NOX inhibitors [111]. In an earlier study, mutant SOD1, as present in the familial type of ALS, expressed in human cell lines, was shown to activate NOX directly through the Rac1 regulator protein [112]. The aggregation and accumulation within neurons of mutant ATXN7 because of polyglutamine (poly-Q) expansion has been held responsible for the inherited neurodegenerative disease spinocerebellar ataxia type 7 (SCA7). In a cell-based model of this disease, the expression of mutant ATXN7 is accompanied by increased ROS production, aggregation of ATXN7, and cytotoxicity, which are preventable by a NOX inhibitor [113].

### 2.6. Alzheimer's Disease, Oxidative Stress, and NOX

#### 2.6.1. Oxidative Stress in AD

Alzheimer's disease (AD) is characterized by a diffuse loss of neurons in the hippocampus, enterorhinal cortex, amygdala, and different regions of the neocortex with extracellular deposition of oligomerized amyloid beta peptide (A*β*42) called amyloid plaques and intraneuronal neurofibrillary tangles composed of phosphorylated tau protein [13]. The disease causes progressive dementia and loss of multiple cognitive domains in a devastating form leading to death within 3–9 years of diagnosis. The accumulation of oxidative damage markers of lipids, proteins, and nucleic acids has been shown in multiple studies in CSF or postmortem brain tissue in AD, and the topic is reviewed extensively [13, 87, 88, 93, 94, 114–117]. Such oxidative damage in the brain is also detectable in AD transgenic animals along with the deposition of amyloid plaques [118, 119]. In addition, the accumulation of transition metals like Fe and Cu in the AD brain has been shown in many postmortem studies using histochemical and magnetic resonance spectroscopic methods, but a meta-analysis subsequently has challenged this notion and suggested significant citation bias [120–123]. Antemortem imaging studies have also indicated iron accumulation in AD brains in several areas, but more extensive in vivo studies are necessary [124, 125]. Although ROS can interact with any biomolecule within neurons, oxidative damage to proteins in particular could be important in AD pathogenesis through disruption of neuronal energy metabolism and proteostasis and aberrant redox signalling through activation of stress-activated protein kinases (JNK, p38, and ERK 1/2) or oxidative modifications of redox-sensitive transcription factors [126–128].

Most reviews dealing with oxidative damage in AD have discussed the reasons for enhanced ROS formation in the AD brain. In general, transition metal-catalysed ROS formation, especially with the metal liganded with A*β*, could be an important contributor, and in addition, increased ROS formation can take place from dysfunctional mitochondria which is characteristic of AD [128–131]. On the other hand, the release of ROS from activated microglia as a part of the inflammatory response in the AD brain is also important, and NOX could be playing a crucial role in this process.

#### 2.6.2. NADPH Oxidase and AD

NADPH oxidase activation has been strongly implicated in the pathogenesis of AD as evident from postmortem studies showing the translocation of NOX2 subunits p47^phox^ and p67^phox^ from cytosol to membrane, and this activation presumably takes place in activated microglia [132]. In another study, subjects were grouped on the basis of antemortem behavioural testing and postmortem histopathological assessment as no cognitive impairment (NCI), preclinical AD, mild cognitive impairment (MCI), and early to moderate AD; NOX activity was measured luminometrically, and protein expression levels of NOX2 subunits were assessed by immunoblotting [133]. The NOX enzyme activity was elevated in MCI, and different grades of AD compared to that in NCI. NOX2 subunit (p47^phox^, p67^phox^, and p40^phox^) levels also remained high in different grades of AD, and in addition, a strong inverse correlation was observed with increased NOX activity associated with decreased cognitive functions [133]. In another longitudinal follow-up study of control subjects and patients of preclinical AD, MCI, and advanced AD, increased NOX activity was seen in the temporal cortex of MCI patients but not in those of preclinical AD or advanced AD subjects; immunohistochemical and immunoblotting analyses showed increased levels of gp91^phox^ and p47^phox^ in the MCI group [134]. Further, this study showed that gp91^phox^ was expressed in microglial cells as well as in neurons, and the toxic action of soluble oligomeric A*β*42 on neurons in culture were diminished by the NOX inhibitor apocynin [134]. The enhanced activity of NOX2 in AD may be caused by the activation of microglia by A*β* which releases ATP which in turn leads to NOX2 activation and ROS production; the process is mediated through the activation of the purinergic receptor P2X7 and requires Ca^2+^ influx [135]. Another earlier study showed that microglia in primary culture stimulated by ATP acting through the purinergic receptor P2X7 release superoxide radical (O_2_^**•-**^), and this process is mediated by NOX activation [136]. Furthermore, such activated microglia can lead to neuronal death in coculture suggesting a clear link among neuroinflammation, NOX activation, oxidative stress, and neurodegeneration [136]. Intracerebrovascular injection of LPS or A*β* oligomers causes an inflammatory response through the activation of microglia, but this response is inhibited in NOX-deficient (p47^phox^ or gp91^phox^ deficient or apocynin treated) mice where the microglia attain the alternative phenotype (M2) responsible for tissue healing and repair [137]. Likewise, neuroblastoma cells with an overexpression of APP (wild-type or containing multiple mutations of familial AD) degenerate when cocultured with microglial cells because of ROS production by NOX, and this is expectedly attenuated by the NOX inhibitor DPI or radical scavengers [138].

**Figure 1:**
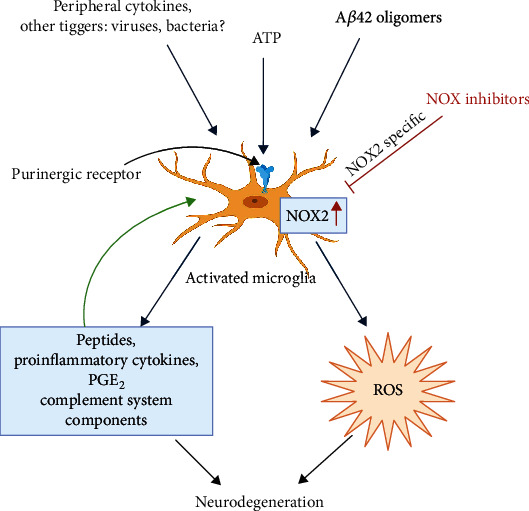
NOX activation in brain microglia in Alzheimer's disease. The microglial NOX2 is upregulated through multiple triggers including A*β*42. Proinflammatory cytokines, chemokines, and NOX2-derived ROS act in concert to cause further activation of microglia and also neuronal damage linking inflammation and oxidative damage in AD pathology. The potential beneficial effects of NOX2 inhibitors have been indicated (image was created using BioRender).

In addition to NOX2, high levels of NOX1 and NOX3 mRNA have been observed in the frontal lobe of AD patients (early stages), indicating the contribution of other NOX isoforms in AD neuropathology [139]. The activity of NOX and the expression of the NOX4 subunit are also consistently elevated in the brain of APPxPS1 knock-in mice with a significant linear correlation between NOX activity and the age-dependent accumulation of A*β* with cognitive dysfunction [140]. On the other hand, transgenic mice (Tg2576) carrying human APP with the Swedish mutation and lacking in the catalytic subunit of NOX2 fail to develop oxidative damage, neurovascular dysfunction, and cognitive deficits at 12-15 months of age unlike Tg2576 mice with intact NOX2, though the brain amyloid beta burden in both the groups were similar [141]. Thus, a mounting body of evidence demonstrates NOX activation and ROS generation in the AD brain and primarily implicates microglial response as the trigger for increased ROS production. In the AD brain, soluble oligomers of amyloid beta peptide and other inflammatory triggers activate the microglia to M1 state with changes in morphology and exhibition of many inflammatory surface markers [128, 142, 143]. The microglia proliferate and assemble near the amyloid plaques releasing many peptides, proinflammatory cytokines, chemokines, prostaglandins like PGE_2_, components of the complement system, and ROS, and thus a strong inflammatory response is generated which is partly responsible for neuronal and synaptic degeneration [128, 142–144]. The increased ROS production in reactive microglia occurs from activation of NOX2; ROS can cause degeneration of neurons and through redox-signalling pathways enhance the formation and release of proinflammatory cytokines from the microglia [145]. This suggests an overlap of oxidative damage pathways, redox-signalling mechanisms, and inflammatory response in AD brain.  Figure 1 summarizes the mechanistic processes linked to NOX activation in microglia and the potential benefits of NOX2 inhibitors.

## 3. NADPH Oxidase Inhibitors in AD

It is apparent from the discussion above that NOX inhibitors will have substantial effects on the pathogenesis of AD, attenuating both oxidative stress and neuroinflammation in the brain; thus, they are potential candidates as disease-modifying agents in AD. Furthermore, NOX2, present predominantly in microglia, appears to be the most important isoenzyme involved in AD pathogenesis; thus, a specific inhibitor of NOX2 instead of a general NOX inhibitor could be even more effective. The NOX inhibitors are comprised of a heterogeneous group of molecules that include both peptide inhibitors and small molecule inhibitors. The peptide inhibitors are designed to prevent the binding of cytosolic accessory proteins to the membrane-bound catalytic portion of NOX; thus, they act as specific inhibitors of different isoenzymes of NOX (NOX2-ds-TAT and NoxA1ds are specific for NOX2 and NOX1, respectively) [146]. Many small-molecule inhibitors of NOX are currently available for research applications, and some of them are also being tested in clinical trials [146–148].

The classification of NOX inhibitors is complex. The older generation of small molecule NOX inhibitors include diphenylene iodonium (DPI) and apocynin, and the relatively less tested 4-(2-aminoethyl)-benzenesulphonyl fluoride (AEBSF) or plumbagin, while the more recently introduced compounds are GLX351322 and GSK2795039. A major issue with these drugs is the presence of additional actions such as direct ROS-scavenging properties, rho kinase inhibition, flavoprotein inhibition, and serine kinase inhibition. Many of them do not directly inhibit NOX but instead act on upstream triggers and downstream pathways of NOX [26, 148]. Many of these drugs are not specific for any particular isoenzyme of NOX, but GKT137831 and GKT36901 are claimed to be specific for NOX1 and NOX4 [148]. This not only makes it difficult to judge how much their actual therapeutic benefits may be ascribed to NOX inhibition but also results in off-target effects, making clinical use difficult [26, 148]. One drug that has been prominently tested, especially in a variety of cardiovascular disease states in animal models and some clinical trials is apocynin. It is a phenolic compound which is derived from the medicinal plant *Jatropha multifida* [149]. It has been also suggested that apocynin, which mainly inhibits NOX2, may have a beneficial role in Alzheimer's disease. A study investigating apocynin at an oral dose of 10 mg/kg daily in a hAPP (751) SL transgenic mouse model of AD found a significant reduction of plaque size within the cortex and hippocampus and a reduction of microglia number in the cortex [150]. In another experiment, male Wistar rats treated with scopolamine to induce an AD phenotype showed improvement in cognitive test performance when administered apocynin. This was accompanied by a decline in amyloid *β* concentration in the rat hippocampi and a decrease in superoxide anion concentration [151]. Apart from the traditional small molecule inhibitors, several new agents such as GKT136901 and GKT137831; ML171; VAS2870 and VAS3947; S17834; Fulvene-5; the triphenylmethane derivative dyes, namely, imipramine blue, brilliant green, and Gentian violet; grindelic acid; ebselen; perhexiline; and Shionogi I and II have been proposed to have NOX-specific inhibitory activity based on experimental studies [26, 152]. Most of these agents, however, have not made it to the clinical trial stage, at least in neurodegenerative disorders. The focus of clinical studies in Alzheimer's disease thus remains on naturally obtained compounds with not only NOX inhibitory but also other beneficial effects. These include berberine, blueberry-derived polyphenols, relatively NOX-specific celastrol, EGCG derived from green tea, *Gingko biloba*, resveratrol, and others.  Table 1 summarizes several human studies of these agents in patients with Alzheimer's disease and its precursor states such as mild cognitive impairment [153–163]. It is evident that the results are quite variable, and hence, further studies are warranted to evaluate their role in AD therapeutics. It is also to be understood that NOX-generated ROS take part in redox signalling under physiological conditions in the brain; thus, inhibition of NOX as a therapeutic measure may be fraught with additional problems.

## 4. Conclusion

The complexity of AD pathogenesis especially in sporadic cases precludes any “magic bullet” approach towards the therapy of this disease. Multiple disease-modifying drugs and multitargeted drugs could become important in the coming days to combat AD. To that extent, NOX inhibitors may provide an “add-on” therapy to maximize clinical benefit in AD treatment. However, a molecule specifically and directly inhibiting NOX2 without “off-target” effects would be necessary, and in addition, its bioavailability in the CNS and long-term toxicity have to be carefully evaluated.

## Figures and Tables

**Table 1:** Drug trials of NOX inhibitors in Alzheimer's disease and its precursor conditions.

Country, trial code, reference if published	Sponsor	Phase	Diagnosis	Study design	Sample size and age	Treatment	Outcome	Duration	Results and status
Germany NCT00951834	Charité University, Berlin	2, 3	Early stage AD	Randomized, placebo controlled	*N* = 21, ≥60 years	EGCG (200-800 mg) as an add-on to donepezil	(1) ADASCog score (2) Safety, MMSE, brain atrophy, time to hospitalization, time to death, and others	18 months	Results not posted

Spain NCT03978052	Parc de Salut Mar	NA	Apo E4 carriers with SCD	Randomized, double blind, personalized, placebo controlled, four-arm trial	*N* = 200, 60-80 years	Multimodal intervention (diet, physical activity and cognitive activity) and EGCG (5-6 mg/kg up to 520 mg/day)	(1) ADCS-PACC-like score (2) Changes in functional neuronal connectivity tested by fMRI, changes in structural connectivity networks	12 months of treatment; 24 months total study duration	Ongoing

Spain NCT01699711 [153]	Parc de Salut Mar	2	DS neurological disease	Randomized, double blind, placebo controlled	*N* = 87, 14-29 years	EGCG (9 mg/kg) and cognitive training	(1) Change in cognitive evaluation and amyloidosis biomarkers (2) Change in DYRK1A activity biomarkers, lipid oxidation biomarkers, neurophysiology, neuroimaging, and others	12 months	EGCG better than placebo in improving visual recognition memory, inhibitory control and adaptive behaviour

US NCT01504854 [154]	ADCS, National Institute on Aging	2	Mild-moderate AD	Randomized, double blind, placebo controlled	*N* = 119, ≥50 years	Oral resveratrol (500 mg/day; increased up to maximum 2 g/day)	(1) Number of adverse events, volumetric MRI brain changes from baseline (2) Change in ADCS-ADL, CSF-A*β*40 levels	52 weeks	Nausea, diarrhoea, weight loss common with resveratrol. CSF and plasma A*β* declined more in placebo group. Brain volume loss and ventricular volume increase more in resveratrol group.

US NCT00678431 [155]	US Dept. of Veterans Affairs	3	Probable AD patients with MMSE 12-26	Randomized, double blind, placebo controlled	*N* = 27, 50-90 years	Oral liquid resveratrol, glucose and malate	(1) ADASCog (2) ADCS-CGIC	12 months	All outcome scores showed less deterioration in treatment group; however, statistically insignificant

US NCT02502253	Johns Hopkins University, Icahn School of Medicine at Mount Sinai	1	Amnestic MCI; impaired fasting glucose or clinically stable type 2 diabetes	Randomized	*N* = 48, 50-90 years	BDPP, low-, moderate-, high-dose study	Adverse events and serious adverse events, CSF penetration of BDPP, effect on mood, and effect on cognition	4 months	Recruiting

Turkey NCT04044131	Istanbul Medipol University Hospital, ScandiBio Therapeutics AB, and others	2	Mild to moderate AD (ADASCog ≥ 12 and CDR ≤ 2)	Randomized, double blind, placebo controlled	*N* = 60, >50 years	Mixture of NAC, carnitine, nicotinamide riboside, and serine (metabolic cofactors)	(1) MMSE, ADASCog, ADCS-ADL (2) Volumetric brain MRI, resting state fMRI, NPI, MOCA, serum omics, microbiota, adverse events, and biochemical monitoring	3 months	Recruiting

US NCT01320527 [156, 157]	University of Massachusetts, Worcester	2	AD and MCI	Randomized, double blind, placebo controlled	*N* = 106, ≥40 years	NF having folic acid 400 *μ*g, vitamin B12 6 *μ*g, vitamin E 30 IU, SAM 400 mg, NAC 600 mg, acetyl-L-carnitine 500 mg	(1) Cognitive improvement by CLOX-1 and DRS (2) Improvement in NPI and ADL	12 months; first assessment at 3 months	Statistically significant improvement in the NF group versus placebo in cognitive assessment by CLOX-1 and DRS. Nonsignificant improvement in NPI and ADL. Continuation as open-label in 24 patients and evaluated at 12 months; participants maintained baseline cognitive performance and BPSD.

US NCT01370954	Pamlab, Inc. and InfoMedics, Inc.	NA	Early memory loss, MCI, AD, and VD	Prospective observational	*N* = 204, 50-80 years	Medical food CerefolinNAC® having NAC 600 mg, methyl cobalamin 2 mg, L-methyl folate calcium 6 mg	(1) QOL-AD measure of quality of life (2) Overall patient satisfaction	3 months	Results not posted

US NCT02033941	Hillel Grossman, NCCIH	2	Probable AD with MMSE score of 12-26	Randomized, double blind, placebo controlled	*N* = 20, all ages	Grape seed polyphenolic extract	(1) Pharmacokinetic analysis, CSF tau and phosphorylated tau protein, adverse events (2) A*β* in plasma and CSF, scores on ADASCog, ADCS-CGIC, MMSE, ADL	22 months	Recruiting

China NCT03221894	Dongzhimen Hospital, Beijing	NA	AD (mild-severe on MMSE)	Observational study	*N* = 90, 50-85 years	GRAPE granules (having herbal medicines such as ginseng, *Curcuma*, *Acorus*, *Polygala*, and berberine)	(1) MMSE (2) ADL, NPI, and CDR	12 months	Results not posted, status as of 2017 was recruiting

South Korea NCT00391833	Seoul National University Hospital	1, 2	AD	Observational randomized, open label	*N* = 97, 40-83 years	*Panax ginseng* powder 4.5 g/day	MMSE and ADASCog scores	12 weeks therapy; assessment at 12 weeks and after 12 weeks of discontinuation of therapy	Statistically significant improvement in MMSE and ADASCog scores between the groups at 12 weeks. Improvement dissipated at 24 weeks (after 12 weeks of ginseng discontinuation) and adverse events were seen in 12% of patients treated with ginseng and 15% of the control group. Dizziness, headache, diarrhoea, and anorexia were the common adverse events seen in both groups.

Hong Kong NCT00164749	Chinese University of Hong Kong, BUPA Foundation, Kwong Wah Hospital	1, 2	AD	Randomized, double blind, placebo controlled	*N* = 34, ≥50 years	Curcumin powder or capsule (4 g or 1 g) along with standard treatment of ginkgo leaf extract 120 mg/d in all groups (including placebo)	(1) Plasmaisoprostanes, serum A*β*_40_ (2) Change in cognitive function (MMSE score), curcumin and metabolites in plasma	6 months (some variables at 1 month)	Cognitive scores did not improve with curcumin. Vitamin E increased over 1 month with curcumin. Serum A*β*_40_ did not change.

India NCT01001637	Jaslok Hospital and Research Center, others	2	AD, MMSE score of 5-20	Randomized, double blind, placebo controlled	*N* = 26, 50-80 years	Solid lipid curcumin particle (SLCP) formulation	(1) Mental capacity (based on tests) (2) Blood concentration of A*β*	2 months	Results not posted

US NCT00099710 [158]	John Douglas French Foundation	Phase 2	Mild-moderate AD	Randomized, double blind, placebo controlled for 6 months followed by open label for next 6 months	*N* = 30, ≥50 years	Curcumin C3 complex (2 g or 4 g daily)	(1)Adverse events, ADASCog, changes in clinical laboratory tests (2) NPI, ADCS-ADL, plasma A*β*, CSF isoprostanes, t-tau, p-tau, and A*β*	6 months	No difference in clinical efficacy or biomarkers. Clinically insignificant increase in blood glucose and decrease in hematocrit in curcumin group. GI symptoms occurred in 12.5% patients of curcumin group leading to withdrawal from study.

US NCT01811381	Veterans Affairs Office of Research and Development	2	MCI, MMSE > 24	Randomized, double blind	*N* = 80, 50-90 years	Curcumin; aerobic and anaerobic yoga/exercises	(1) Blood biomarkers: TNF*α*, N-terminal BNP, IL-6, IL-1*β*, VCAM-1, ApoE, etc. (2) NPI, adverse events, 18-FDG-PET, FAQ	12 months	Active, not recruiting

US NCT01716637	Life Extension Foundation Inc.	1	AD (NINCDS-ADRDA criteria)	Open label, crossover	*N* = 12, 60-85 years	Perispinal etanercept injection subcutaneously and dietary supplements having curcumin, quercetin, resveratrol, *ω*-3 fatty acids	(1) MMSE score (2) ADASCog score, MOCA score	16 weeks	Results not posted

France NCT00814346	Ipsen	2	Three groups: mild AD; cognitively normal elderly; cognitively impaired elderly (MMSE-20-28 for AD)	Randomized double blind, placebo controlled followed by open label	*N* = 49, ≥65 years	EGb761® Ginkgo (120 mg twice daily)	(1) Change in brain glucose metabolism (18-FDG-PET) at 1 month (2) CDR, MMSE, GDS, MMSE, adverse events in memory complaint/normal group	18 months	(1) Not reported (2) Falls occurred in 12%; constipation, insomnia, and depression occurred in 7.3% each; gastrooesophageal reflux, vertigo, and dyspnoea occurred in 4.8% each, in the open phase

China NCT03090516	The First Affiliated Hospital with Nanjing Medical University	2, 3	Mild-moderate AD	Randomized	*N* = 240, 50-85 years	Donepezil versus donepezil plus ginkgo versus ginkgo	MMSE score, EEG, MRI, ADASCog score, LFT, RFT, NPI, and ADL	3 months	Recruiting as of August 2019

US NCT00010803 [159–161]	NCCIH, others	3	Normal cognition and MCI patients	Randomized, double blind, placebo controlled	*N* = 3069, ≥75 years	Ginkgo (EGb761®) 120 mg twice daily	(1) All cause dementia including AD (2) CVD events or mortality, progression of cognitive decline	8 years	Ginkgo had no effect on decreasing dementia, cognitive decline, and cardiovascular events. More PVD events were seen in placebo group.

France NCT00276510 [162]	Ipsen	3b/4	Patients with memory complaints	Randomized, double blind, placebo controlled	*N* = 2854, ≥70 years	Ginkgo (EGb761®) 120 mg BD	(1) Conversion to AD (2) Concomitant diseases, safety, rate of cognitive abilities decline	5 years	Ginkgo had no effect on decreasing AD, overall deaths and stroke. No difference in safety profile.

US NCT00042172	University of Iowa, National Institute of Mental Health	4	Patients with MCI and subjective memory complaints	Randomized	*N* = 40, ≥65 years	Donepezil versus placebo for 6 months then donepezil plus ginkgo versus donepezil alone for next 6 months	Brain blood flow using PET	12 months	Results not posted

US NCT01009476	Janssen-Cilag G.m.b.H	NA	Mild to moderate AD/mixed dementia	Prospective observational, noninterventional	*N* = 1134, ≥50 years	Galantamine or nootropics (*Ginkgo*, piracetam, nicergoline, etc.)	Cognitive decline, safety, vital functions, caregiver's burden, etc.	12 months	Results not posted

US, Israel, UK NCT00940589	Neurim Pharmaceuticals Ltd.	2	Mild-moderate AD (MMSE score > 15)	Randomized, double blind, placebo controlled	*N* = 73, 50-85 years	AChase inhibitor and melatonin (prolonged release) 2 mg versus AChase inhibitor and placebo	(1) ADASCog change (2) iADL change, MMSE change	6 months	Nonsignificant change in ADASCog between the groups. iADL improved significantly (*P* < 0.05) in placebo compared to melatonin (1.62 versus 0.77). MMSE declined less in the melatonin group (-0.3 versus -1.9). Adverse events: gastrointestinal seen in 28.2% of the melatonin group versus 14.7% of the control group; respiratory disorders seen in 20.5% of the melatonin group versus 11.7% of the control group. Angina, falls seen only in the melatonin group (7.7% each); increased blood sugar in the melatonin group (5%) versus the control group (2.9%). Neuropsychiatric disorders common in the melatonin group (17.9%) versus the control group (14.7%).

US NCT00000171 [163]	National Institute on Aging (NIA)	3	AD, MMSE ≤ 26, dyssomnia	Randomized, double blind, placebo controlled	*N* = 157, ≥55 years	Melatonin 2.5 mg SR, melatonin 10 mg IR	(1) Change in nocturnal sleep time (2) Awake period, daytime agitation, change in ADASCog, MMSE, HAM-D	8 weeks	No significant change in objective sleep outcomes. Caregiver rating of sleep quality better in 2.5 mg SR melatonin versus placebo. Adverse events similar between the groups

US NCT03954899	NazanAksan, University of Iowa	NA	MCI, MOCA score ≥ 18	Randomized, double blind, placebo-controlled study assessing disease-modifying role of melatonin	*N* = 230, 60-80 years	Melatonin 5 mg	(1) Episodic memory (2) Overall cognitive function, CSF-p-tau, t-tau, A*β*42, sleep efficiency, and others	44 weeks	Recruiting

Abbreviations: AChase = acetylcholine esterase; AD = Alzheimer's disease; ADASCog = Alzheimer's Disease Assessment Scale—cognitive subscale; ADCS = Alzheimer's Disease Cooperative Study; ADCS-CGIC = Alzheimer's Disease Cooperative Study—Clinical Global Impression of Change; ADCS-PACC = Alzheimer's Disease Cooperative Study—Preclinical Alzheimer Cognitive Composite; ADL = activities of daily living; Apo E = apolipoprotein E; A*β*40 = amyloid beta 40; BDPP = bioactive dietary polyphenol preparation (has grape seed polyphenolic extract and resveratrol); BNP = brain-type natriuretic peptide; BPSD = behavioural and psychological symptoms in dementia; CDR = clinical dementia rating; CSF = cerebrospinal fluid; CVD = cardiovascular disease; DRS = Dementia Rating Scale; DS = Down's syndrome; DYRK1A = dual-specificity tyrosine phosphorylation-regulated kinase-1A; EEG = electroencephalogram; EGCG = epigallocatechin gallate; FAQ = Functional Activities Questionnaire; FDG = fluorodeoxyglucose; fMRI = functional magnetic resonance imaging; GDS = Geriatric Depression Scale; HAM-D = Hamilton Depression Rating Scale; iADL = instrumental activities of daily living; IL = interleukin; LFT = liver function test; MCI = mild cognitive impairment; MMSE = Mini Mental State Examination; MOCA = Montreal Cognitive Assessment; NA = not applicable; NAC = N-acetyl cysteine; NCCIH = National Center for Complementary and Integrative Health; NINCDS-ADRDA = National Institute of Neurological and Communicative Disorders and Stroke-Alzheimer's Disease and Related Disorders Association; NPI = neuropsychiatric inventory; NF = nutraceutical formulation; PET = positron emission tomography; p-tau = phosphorylated tau protein; PVD = peripheral vascular disease; QOL = quality of life; RFT = renal function test; SCD = subjective cognitive decline; TNF*α* = tumor necrosis factor *α*; t-tau = total tau protein; VCAM-1 = vascular cell adhesion molecule-1; VD = vascular dementia.

## Data Availability

This being a review article, no data was generated during the preparation of this manuscript.
